# Absence of Bladder Outlet Obstruction Is an Independent Risk Factor for Prostate Cancer in Men Undergoing Prostate Biopsy

**DOI:** 10.1097/MD.0000000000002551

**Published:** 2016-02-18

**Authors:** Luigi Cormio, Giuseppe Lucarelli, Oscar Selvaggio, Giuseppe Di Fino, Vito Mancini, Paolo Massenio, Francesco Troiano, Francesca Sanguedolce, Pantaleo Bufo, Giuseppe Carrieri

**Affiliations:** From the Department of Urology (LC, GL, OS, GDF, VM, PM, FT, GC) and Renal Transplantation, University of Foggia, Foggia, Italy; Department of Emergency and Organ Transplantation (GL), University of Bari, Bari, Italy; and Department of Pathology (FS, PB), University of Foggia, Foggia, Italy.

## Abstract

The purpose of this study was to investigate the relationship between bladder outlet obstruction (BOO) and the risk of being diagnosed with prostate cancer (PCa).

Study population consisted of 2673 patients scheduled for the first prostate biopsy (PBx). All patients underwent uroflowmetry before PBx; those with a peak flow rate (PFR) <10 mL/s were considered to have BOO.

The incidence of PCa was 41.3% (1104/2673) in the overall population and 34.1% (659/1905) in patients with serum prostate-specific antigen (PSA) ≤ 10 ng/mL. Univariate and multivariate logistic regression analyses showed that patients with BOO had a significantly (*P* < 0.0001) lower risk than those without BOO of being diagnosed with PCa (33.1% vs 66.9% in the overall population; 30% vs 70% in patients with PSA ≤ 10 ng/mL). As the presence of BOO was significantly correlated to a large prostate volume, another independent predictor of PBx outcome, we tested whether these parameters could be used to identify, in the subset of patients with PSA≤10 ng/mL, those who could potentially be spared from a PBx. If we would have not biopsied patients with BOO and prostate volume ≥60 mL, 14.5% of biopsies could have been avoided while missing only 6% of tumors. Only 10% of the tumors that would have been missed were high-risk cancers.

In conclusion, in men undergoing PBx, the absence of BOO, as determined by a PFR ≥10 mL/s, is an independent risk factor for PCa. Our study provides ground for this simple, noninvasive, objective parameter being used, alone or in combination with prostate volume, in the decision-making process of men potentially facing a PBx.

## INTRODUCTION

Prostate biopsy (PBx) is the standard method for diagnosing prostate cancer (PCa) but the diagnostic yield of this procedure remains low. In current clinical practice the cancer detection rate of a first extended PBx prompted by an elevated serum prostate-specific antigen (PSA) level and/or an abnormal digital rectal examination (DRE) is in the range of 40% and has not been significantly improved by nomograms combining PSA with other readily available clinical characteristics (age, DRE findings, prostate volume, etc).^1,2^ A diagnostic and prognostic role has been evaluated for novel biomarkers such as the precursor isoform [-2]proPSA (p2PSA), Prostate Cancer Antigen 3 (PCA3), and cancer metabolism-related proteins.^3–8^ The introduction of these new circulating markers have improved but not dramatically the accuracy of PCa diagnosis and prognosis. Multiparametric magnetic resonance imaging (mMRI) of the prostate has been suggested to improve PBx diagnostic yield but the optimal clinical application of this imaging technique remains under investigation.^9^ Moreover, diagnostic tools such as mMRI are expensive and/or invasive, thus not easily applicable to everyday clinical practice. In this scenario, the identification of cheap and readily available predictive tools that can be used either alone or in association with other markers represents a major clinical issue.

The potential relationship between bladder outlet obstruction (BOO) and increased serum PSA has been pointed out by Laniado et al.^10^ They have shown that high PSA levels in patients with lower urinary tract symptoms (LUTS) are significantly associated with BOO, resulting in 89% of patients with PSA > 4 ng/mL having BOO due to benign prostatic obstruction (BPO). It has subsequently been shown that patients with minor or no LUTS undergoing PBx because of an elevated serum PSA and being diagnosed with benign prostate all had BOO on pressure uroflowmetry.^11^

A definite diagnosis of BOO requires a pressure-flow study but, given the invasivity of this test, the initial objective evaluation of BOO relies on uroflowmetry. More than 90% of men with a peak flow rate (PFR) of <10 mL/s have BOO.^12^

Given the correlation between BOO and increased PSA levels, the present study aimed to determine whether BOO, as assessed by uroflowmetry, was associated with the risk of being diagnosed with prostate cancer (PCa), in other words whether BOO may be used to predict the outcome of prostate biopsy (PBx).

### Patients and Methods

Data of patients scheduled for ultrasound-guided transrectal PBx because of increased serum PSA (≥4 ng/mL) and/or abnormal digital rectal examination (DRE) were prospectively entered into our institutional review board approved database. All patients underwent PSA measurement before DRE and transrectal ultrasound (TRUS). Uroflowmetry was carried out before PBx, waiting for the patient to report a strong sensation to void. Following local anaesthesia,^13,14^ TRUS was used to determine prostate volume and to guide transrectal prostate sampling according to our systematic 18-core biopsy scheme.^15^

Men receiving medical therapy known to affect PSA levels, or who had previously undergone PBx or invasive treatment for benign prostatic hyperplasia, or with dwelling urethral catheters, or with a voided volume of <150 mL were excluded from the present case-control study.

Two senior uropathologists blind to uroflowmetry data evaluated the specimens according to contemporary diagnostic criteria for high-grade prostatic intraepithelial neoplasia (HGPIN), atypical small acinar proliferation (ASAP) of prostate, and PCa. Patients diagnosed with HGPIN or ASAP were excluded from the present analysis.

The study protocol was approved by University of Foggia Ethics Committee and it conforms to the provisions of the Declaration of Helsinki. Written informed consent to take part was given by all participants.

### Statistical Analysis

Clinical characteristics were considered continuous variables and reported as medians; those with normal distribution, according to the Skewness and Kurtosis test, were compared by Student's *t* test for paired or unpaired data, whereas those with a nonparametric distribution were compared by the Mann–Whitney *U*-test for independent groups. The Spearman correlation was used to evaluate the association between 2 variables, whereas frequencies were compared by the *χ*^2^ test. The combined predictive effect of the covariates was tested by logistic regression analysis, performing a backward selection procedure with a removal criterion of *P* > 0.10 based on the likelihood ratio test. Model calibration was measured by the Hosmer–Lemeshow goodness of fit test. A 2-sided *P* < 0.05 was considered statistically significant. Statistical calculations were carried out using the MedCalc 9.2.0.1 (MedCalc software, Mariakerke, Belgium) and PASW 18 software (PASW 18, SPSS, Chicago, IL).

## RESULTS

**FIGURE 1:**
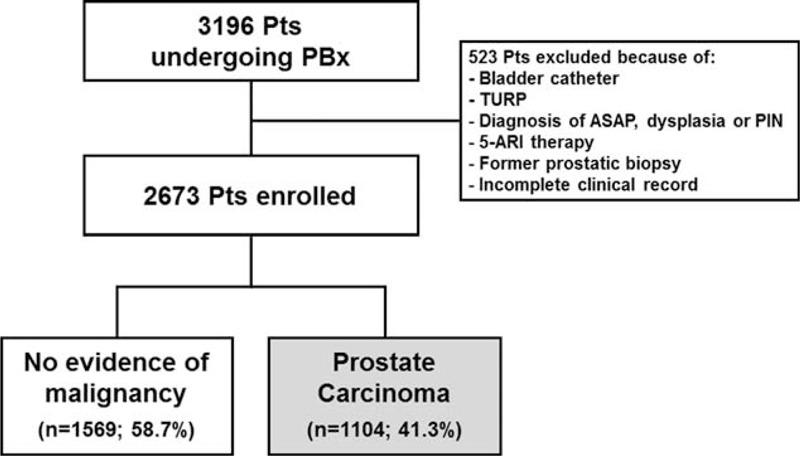
Algorithm showing patient selection and exclusion criteria for the present study. 5-ARI *=* 5-alpha-reductase inhibitor, ASAP *=* atypical small acinar proliferation; PBx *=* prostate biopsy; PIN *=* prostatic intraepithelial neoplasia, TURP *=* transurethral resection of the prostate.

Between January 2006 and December 2014, a total of 2673 patients who underwent transrectal ultrasound-guided PBx at our institution met the inclusion criteria and were enrolled in the present study (Figure 1). Their descriptive characteristics are summarized in Table 1. PCa was found in 1104 patients (41.3%), whereas the remaining 1569 (58.7%) had no evidence of malignancy (NEM). Univariate analysis showed a statistically significant difference in all tested variables (age, PSA, DRE, prostate volume, and PFR) between patients with and without PCa (Table 1). In the subset of patients with PSA levels up to 10 ng/mL; however, there was no significant difference in DRE findings between patients with and without PCa (Table 1). Multivariate logistic regression analysis showed that all variables but DRE were statistical significant predictors of PBx outcome in the overall population as well as in patients with PSA up to 10 ng/mL (Table 2). The Hosmer–Lemeshow statistics confirmed adequate model calibration (*P* = 0.2).

**TABLE 1:**
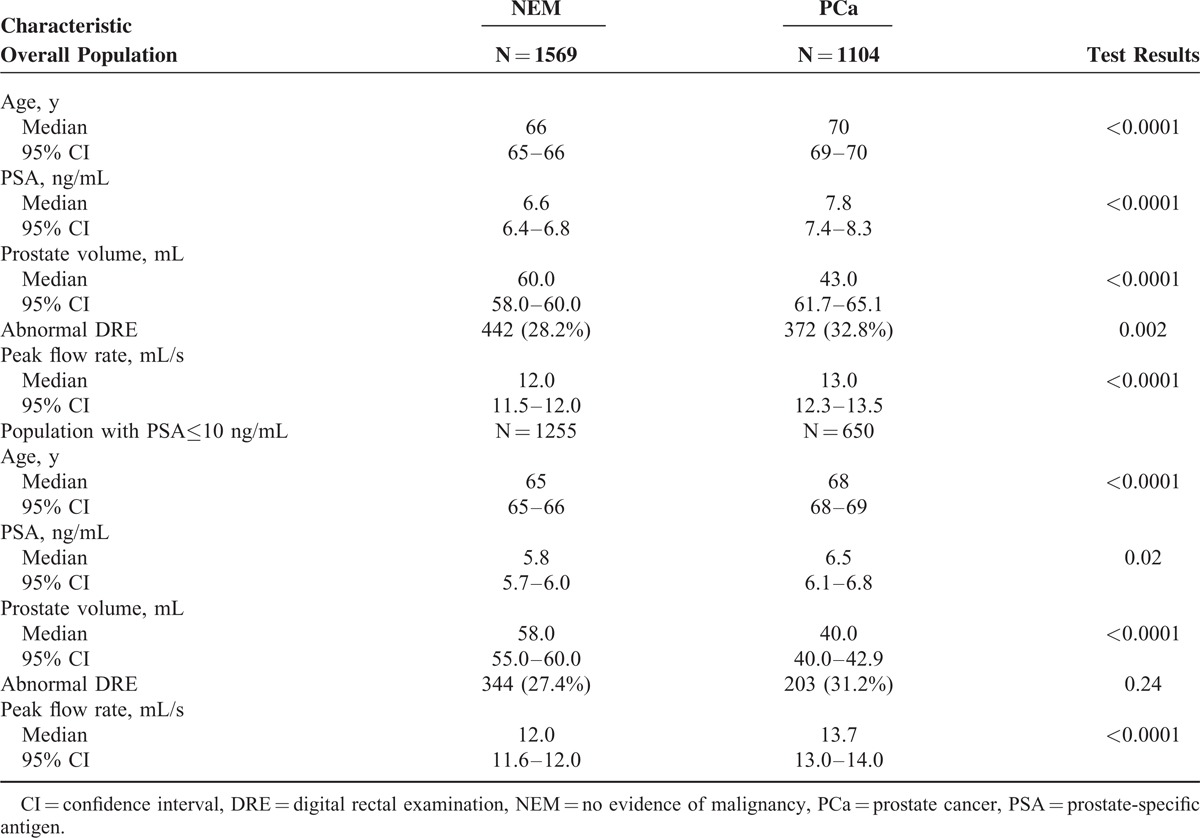
Comparison of Clinical and Pathological Characteristics of Men With No Evidence of Malignancy (NEM) and Men With Prostate Cancer (PCa)

**TABLE 2:**
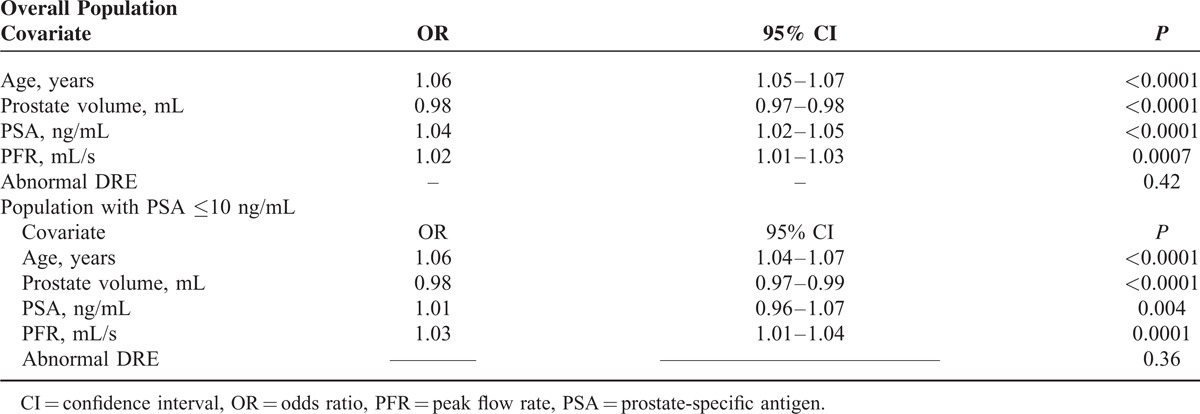
Multivariate Analysis of the Impact of Age, Prostate Volume, Prostate-Specific Antigen (PSA), Peak Flow Rate (PFR), and Abnormal Digital Rectal Examination on Biopsy Outcome

To test the impact of BOO on PBx outcome, patients were stratified according to their PFR value, namely <10 mL/s vs ≥10 mL/s (Table 3). There was a statistically significant difference in age, prostate volume and, most important, in PCa detection rate between patients with and without BOO in both overall population and patients with PSA≤10 ng/mL (Table 3). Interestingly, there was no difference in PSA levels between patients with and without BOO.

**TABLE 3:**
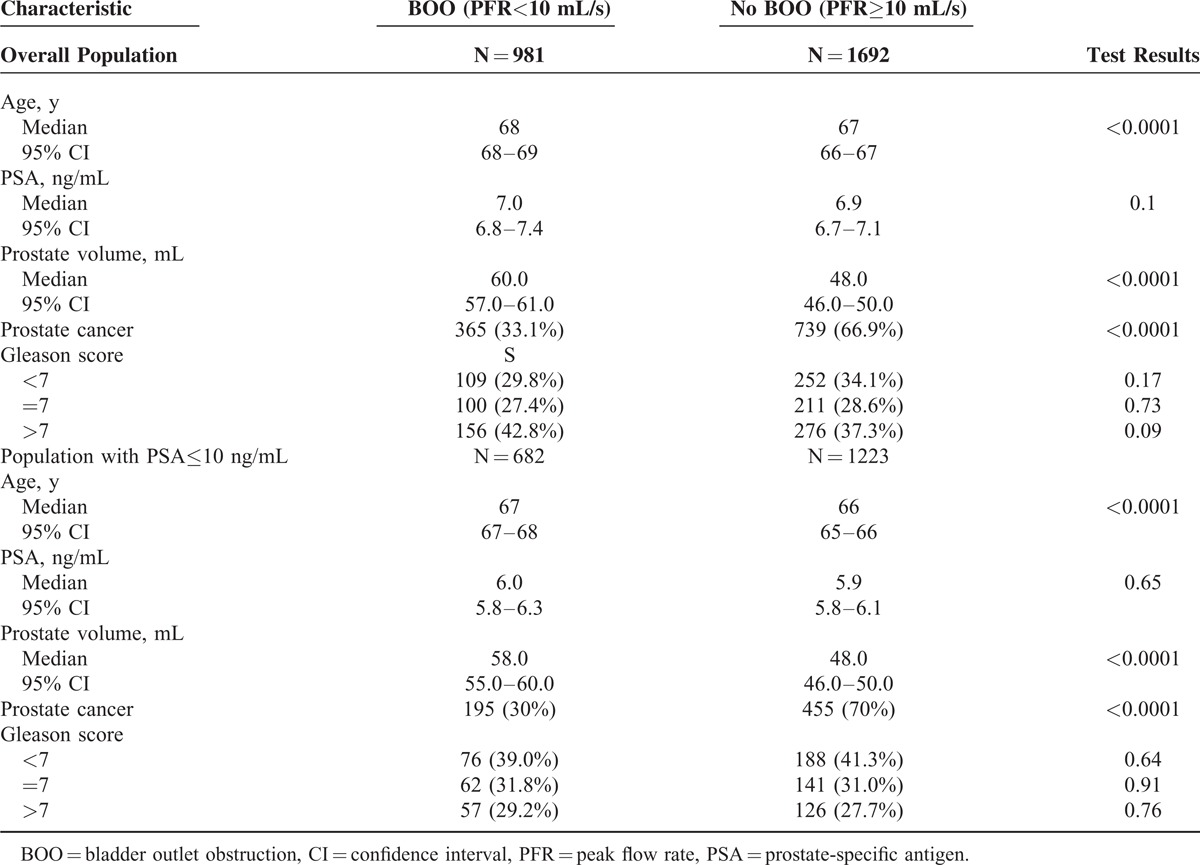
Comparison of Clinical Characteristics and Cancer Detection Rates in Men With or Without Bladder Outlet Obstruction (BOO)

Since prostate volume and PFR were found to be reliable predictors of PBx outcome and to have significant inverse correlation (rs = −0.246; *P* < 0.0001), we tested whether these parameters could be used to identify, in the subset of patients with PSA≤10 ng/mL, those who could potentially be spared from a PBx. Using 60 mL as cutoff for prostate volume (median value in patients with NEM) and 10 mL/s as cutoff for PFR, 14.5% (277/1905) of biopsies could have been avoided while missing only 6% (39/650) of tumors, with only 4 (10%) of the tumors that would have been missed being high-risk (Gleason >7) cancers (Table 4).

**TABLE 4:**
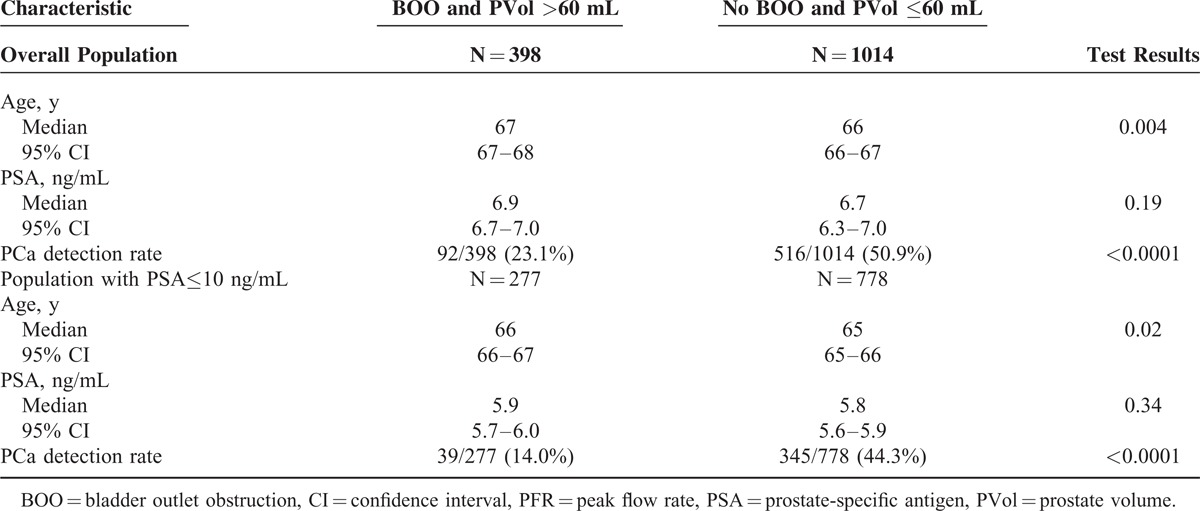
Impact of Bladder Outlet Obstruction (BOO = PFR<10 mL/s) and Large Prostate Volume (PVol>60 mL) on Prostate Cancer Detection Rate

## DISCUSSION

The present study confirmed, by univariate and multivariate analyses, that patients with PCa are older, have higher PSA levels and smaller prostate volume than those without PCa. It also demonstrated that patients with PCa have higher PFR that those without PCa, and this finding occurred in the overall population as well as in patients with PSA ≤ 10 ng/mL. To our knowledge, this finding is novel and strongly supports the hypothesis that BOO, as determined by a PFR<10 mL/s, may increase serum PSA and, by doing so, may lead to potentially unnecessary prostate biopsies.

It is attractive to assume that the increased voiding pressure associated with BOO could lead to urine reflux into prostatic ducts with consequent inflammation and PSA increase. It well known that urine contains different chemical compounds, including uric acid and calcium phosphate crystals, that could be particularly damaging for prostate epithelium. Martinon et al^16^ have shown that these crystals act as danger signal released from dying cells, directly engaging the caspase-1-activating NALP3 (also called cryopyrin) inflammasome, resulting in the production of IL-1β and IL-18. The local production of inflammatory cytokines can increase the influx of inflammatory cells, contributing to prostate inflammation, epithelial damage, and increased release of PSA.

In our experience, the prevalence of PCa was significantly lower in patients with BOO than in patients without BOO (33.1% vs 66.9%; *P* < 0.0001); such difference was even greater (30% vs 70%; *P* < 0.0001) in the subset of patients with PSA ≤10 ng/mL. Interestingly, there was no difference in PSA levels between patients with and without BOO in both overall population and patients with PSA ≤ 10 ng/mL, further supporting the concept that, for the same PSA level, the likelihood of being diagnosed with PCa is much greater for the nonobstructed than for the obstructed patients.

As mentioned above, our study is, to our knowledge, the first to address the relationship between PCa and BOO/BPO as objectively assessed by uroflowmetry. A few studies have previously addressed the potential relationship between PCa and LUTS yielding controversial results. Martin et al^17^ found that the severity of LUTS, as assessed by the validated International Prostate Symptom Score (IPSS), was positively associated with the incidence of localized PCa. However, Matsubara et al^18^ found no difference in IPSS score in men with and without PCa, whereas Porter et al^19^ found that a low American Urological Association (AUA) symptom score was an independent predictor of PCa detection. A large multicenter screening study, whereby 264 of 6630 patients were diagnosed with PCa, revealed that the absence of urinary symptoms predicted PCa; however, no validated symptom score questionnaire was used.^20^ Another large screening study, whereby 2467 out of 65,871 men were diagnosed with PCa, showed that a history of LUTS improved the prediction of an individual's risk for PCa, but also that LUTS among men with a PSA level ≥3 ng/mL were negatively associated with PCa.^21^ Again, no validated symptom score questionnaire was used. Finally, the Gothenburg Randomized Screening Trial,^22^ not using a validated symptom score questionnaire, showed that the absence of voiding symptoms was an independent risk factor for PCa detection, but this inverse association was restricted to men with large (>38 mL) prostates. Moreover, in a recent study of 1780 patients undergoing first PBx, we showed that prostate volume and postvoid residual urinary volume were 2 accurate predictors of biopsy outcome.^23^

For many years there has been a discussion about the reason for the inverse relationship between PCa and prostate volume. Many studies demonstrated that the risk of a large prostate volume to result in a reduced probability of sampling malignant tissue is low.^24,25^ Zackrisson et al found that, among 456 men with 1 set of benign PBx and who underwent 2 additional sets of 18-core PBx, in no cases of prostates with volume of <20 mL, either PCa or PSA normalization was found.^24^ Similarly, van Leeuwen et al found that, among 1305 men with an initial benign PBx, those with smaller prostates and high PSA values had, in the following 8 years, an increased risk of PCa detection and of a more aggressive tumors.^25^

Our study confirms the inverse relationship between PCa and prostate volume as well as the inverse correlation between prostate volume and PFR. Taking these findings together, there are grounds to assume that large prostate volume resulting in BOO may falsely increase PSA levels thus leading to unnecessary PBxs. As a matter of fact, our study showed, in agreement with the Gothenburg Randomized Screening Trial,^22^ that patients with large prostate volume and BOO had a significantly lower risk of being diagnosed with PCa. To turn such findings into clinical practice, we tested whether these 2 objective clinical parameter, PFR and prostate volume, could play a role in the decision-making process of candidates to PBx because of an increased serum PSA level. If we would have not biopsied patients with BOO (PFR <10 mL/s) and large prostate volume (>60 mL, the median value in patients with NEM), 14.9% (398/2673) PBxs would have avoided while missing 8.3% (92/1104) PCas, including 41 high risk cancers. In the subset of patients with PSA≤10 ng/mL, 14.5% (277/1905) of PBxs could have been avoided while missing 6% (39/650) of PCas; only 4 (10%) of the PCas that would have been missed were high-risk cancers.

The strengths of our study include its prospective nature, the use of a standardized extended PBx scheme as well as a standardized protocol for PFR measurement, and pathologists being blind to uroflowmetry data. Potential study limitations include single measurement of PFR, although this test is widely considered accurate, study population consisting of white men only, thus limiting findings applicability to other ethnicities, and lack of external pathological review. In addition, it should be pointed out that in this study we used a TRUS-guided biopsy with an extended 10-core biopsy scheme as previously described.^13,14^ Although we have shown that the addition of 4 lateral peripheral samples did not increase the cancer detection rate of the 10-core biopsy scheme,^15^ it is well known that with this approach it exists the possibility of undersampling the prostate, especially in the anterior and apical areas. These regions are well recognized as being difficult to sample with a transrectal approach.^26,27^ In recent years, the introduction of active surveillance as option for patients with very low-risk PCa has led to the identification of a subset of patients who should be considered for further biopsy, as their pathology might be aggressive. In this scenario, an emerging entity is represented by the prostatic evasive anterior tumor syndrome (PEATS), a condition characterized by combination an aggressive and anterior PCa that manifests as rising PSA values in patients with previous negative TRUS-guided biopsy.^28^ These data highlight the importance of performing a PBx using a transperineal approach in high-risk patients with prior negative transrectal biopsy.

## CONCLUSIONS

In men undergoing PBx, the absence of BOO, as determined by a PFR≥10 mL/s, was found to be an independent risk factor for PCa. These findings suggest the PFR to represent a simple, noninvasive, objective parameter to predict biopsy outcome that can easily be used by clinicians, alone or in combination with prostate volume, in the decision-making process of men potentially facing a PBx. Our study also provides grounds for this new parameter being externally validated in other ethnicities as well as being incorporated into more sophisticated prognostic models.
